# Influence of Laser Treatment on the Corrosion Resistance of Cr_3_C_2_-25(Ni20Cr) Cermet Coating

**DOI:** 10.3390/ma14154078

**Published:** 2021-07-22

**Authors:** Mieczyslaw Scendo, Wojciech Zorawski, Katarzyna Staszewska-Samson, Anna Goral

**Affiliations:** 1 Institute of Chemistry, Jan Kochanowski University of Kielce, PL-25406 Kielce, Poland; katarzyna.staszewska@onet.pl; 2 Faculty of Mechatronics and Mechanical Engineering, Kielce University of Technology, PL-25314 Kielce, Poland; ktrwz@tu.kielce.pl; 3 Institute of Metallurgy and Materials Science Polish Academy of Sciences, PL-30059 Krakow, Poland; a.goral@imim.pl

**Keywords:** Al7075 alloy, cold spray, laser spot, cermet coating, corrosion test

## Abstract

The influence of the laser treatment on the corrosion resistance of the Cr_3_C_2_-25(Ni20Cr) cermet coating on the Al7075 (EN, AW-7075) substrate (Cr_3_C_2_-25(Ni20Cr)/Al7075) was investigated. The coating was produced by the cold sprayed (CS) method. The tested coatings were irradiated with a laser spot speed of 600 mm/min, 800 mm/min, and 1000 mm/min. The mechanical properties of the Cr_3_C_2_-25(Ni20Cr)/Al7075 were characterized by microhardness (HV) measurements. The surface and microstructure of the specimens were observed by ascanning electron microscope (SEM) and other assistive techniques. The corrosion test of materials wascarried out by using the electrochemical method in the acidic chloride solution. Cermet coatings perfectly protect the Al7075 substrate against contact with an aggressive corrosion environment. The laser remelting process of the Cr_3_C_2_-25(Ni20Cr) layer caused the homogenization of the structure cermet coatings. The irradiation with the laser beam eliminates microcracks and pores on the Cr_3_C_2_-25(Ni20Cr) surface. However, the best effect of improving the anti-corrosion properties of cermet coating was obtained for the lowest laser spot speed (i.e., 600 mm/min). It was found that the corrosion rate of the Cr_3_C_2_-25(Ni20Cr) cermet coating was reduced by more than two times compared to the highest speed of the laser spot.

## 1. Introduction

Cold sprayed (CS) is the newest method of thermal spraying, which has found a number of unique applications in various industries. CS is a process in which solid powder particles are accelerated over the sonic velocity through a de Lavel nozzle with aconvergent–divergent geometry. Particles have ballistic impingement on a suitable substrate at speeds ranging between 300 and 1200 m/s. In the spraying process, the substrate material does not melt, and the sprayed coating is attached to the substrate mechanically with adhesion, and the connection of coating material is of metallurgical nature. In this case, the particles of material falling on the sprayed surface are flattened and adhering to each other in the form of successive layers and form a continuous coating [1,2]. The main advantages of coatings produced by the cold gas method are high purity and excellent mechanical properties, which are not achievable with the spraying methods used so far [3].

The Al7075 alloy is a high-strength aluminum alloy thatis comparable to many types of steel; it is applicable as aircraft fittings, shafts, gears valve components, and many other structural parts. However, this alloy has lower corrosion resistance than other aluminum alloys. This issue can be solved by the development of the ceramic coating with the improved corrosion resistance of the Al7075 alloy. The modern industry widely applies fabrication of different ceramic coating on metallic substrates to obtain a required property. Usually, these materials are implemented for wear and corrosion resistance. Good adherence of a coating to its substrate is one of the most important aspects of asuccessful coating. Strong bonding between the coating and the substrate has a unique significance when the coated metals are used in an aggressive corrosion environment.

Cermet coating consists of a metal matrix and a hard reinforcing phase. The coating containing chromium carbide particles was distributed in a nickel–chromium alloy matrix, i.e., the Cr_3_C_2_–NiCr system was used for corrosion and wear-resistant applications. The combination of the ceramic and metal phases enables a higher fracture strength to be achieved [4,5]. The Cr_3_C_2_–NiCr cermet coatings obtained in thermal processes [6] were used as anti-corrosion coatings for machine elements exposed to intense wear. They are characterized by increased mechanical properties and are used in industry for their structural integrity and high temperature and wear resistance [7]. Moreover, the use of cermet powders in the form of mixtures ensures better efficiency of the deposition process of the protective layer [8]. In this process, the ceramic particles do not deform but are deposited in the plastic phase of the metal. An advantage of the cold gas spraying process is that the phase composition of the powder in the formed coating can be preserved. The main factors that affect the mechanical properties of the coatings and their microstructure are properly selected parameters of the spraying process, and the morphology of the powder used [9]. On the other hand, the Cr_3_C_2_–NiCr system coatings can be used in corrosion environments at service temperatures up to 800 °C to 900 °C. The corrosion resistance of cermet coatings increases with an increase in chromium carbide in the pre-sprayed powder [10]. The 75Cr_3_C_2_–25NiCr coatings are primarily designed for wear applications either at elevated temperatures or at room temperature in corrosion environments. Moreover, the dense coatings are supposed to provide very good corrosion resistance as compared to porous coatings, as porosities are the preferential corrosion paths through which the corrosion species can penetrate the coatings to reach the substrate and may cause rapid corrosion attack [11,12]. Furthermore, the corrosion resistance of the cermet coatings is also associated with the surface roughness, in a way in which the higher surface roughness, the higher the corrosion attack due to higher surface area [13]. Most often, the coating thickness is approximately 300–500 μm in the case of a cermet coating. However, the thicker coating permits the pass of the electrolyte due to the stresses generated during coating deposition and the corresponding crack formation between different layers [14]. It is worth noting that Cr_3_C_2_–NiCr coatings are characterized by high hardness. Higher values of hardness for the cermet coatings may be due to the high density and cohesive strength of the individual splats as a result of the high impact velocity of the coating particles [11]. On the other hand, the changes in the hardness of the cermet coatings with heat treatment were accompanied by concurrent changes in microstructure (namely the precipitation) of fine carbides and their subsequent growth and recrystallization of the matrix. Moreover, treatment in air generated further hardness increases as a result of internal oxidation of the surface of the cermet coatings [15]. Nowadays, various thermal spray processes, such as detonation-gun (DG), plasma, high-velocity air-fuel (HVAF), or high-velocity oxy-fuel (HVOF) spraying methods, are usedin the production of cermet coatings [16]. However, in the case of the Cr_3_C_2_–NiCr coating in most high-temperature coating processes, complex chemical transformations take place during the coating and the cooling stages [17]. Moreover, in thermal spraying of fine Cr_3_C_2_–NiCr are chemical degradation of the chromium carbides present in the feedstock powder or the reprecipitation and dissolution of the carbide phases into the NiCr matrix [18]; this process can be summarized as:

Cr_3_C_2_ → Cr_7_C_3_ → Cr_24_C_6_ (1)

It seems that using the cold spray method for the production of the Cr_3_C_2_–NiCr coatings can be greatly reduced, such as thermally induced phase reactions and decomposition effects of fine Cr_3_C_2_–NiCr powders. There is still a lack of knowledge about CS spraying of fine Cr_3_C_2_–NiCr carbides, and it is yet unclear to what extent the final phase distribution and the coating crystallite sizes depend on the initial microstructure of the feedstock powder. On the other hand, various methods are used to remove the cermet coating defects, such as heat treatment, sealing, laser remelting, and others [19]. However, the laser remelting of the cermet coating surface is the most recommended because it can be largely eliminated the porosity, providing a more homogeneous and densified microstructure of coatings [20]. It is worth adding that as aresult of laser remelting, the hardness of the cermet coating also increases significantly.

Various methods were used to increase the mechanical and protective properties of metallic coatings. According to the literature reports, the laser treatment method has not been widely used so far. Therefore, laboratory tests were undertaken to determine the effect of laser remelting on the increase in mechanical and anti-corrosion properties of cermet coatings applied to the aluminum alloy substrate.

In the present study, the influence of the laser remelting process on the corrosion resistance of the Cr_3_C_2_-25(Ni20Cr) cermet coatings on the Al7075 substrate was investigated. The cermet coatings were produced by the cold sprayed method. The corrosion test of the materials in the acidic chloride solution (1.2 M Cl^−^) was carried out by using the electrochemical method.

## 2. Materials and Methods

**Figure 1:**
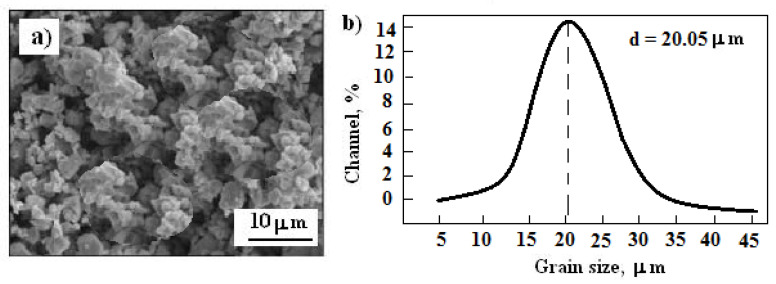
Scanning electron microscopy image: (**a**) structure of the Cr_3_C_2_-25(Ni20Cr) powder, (**b**) powder grain size distribution analysis.

The chemical composition of the Al7075 (EN, AW-7075) alloy is as follows (wt%): 5.6% Zn, 2.5% Mg, 1.6% Cu, 0.22%, Cr < 0.50% are admixtures (i.e., Mn, Fe, and Si), the rest is aluminum. Fine irregular and broken of the Cr_3_C_2_-25(Ni20Cr) (Diamalloy 3004, Oerlikon Metco Inc., Westbury, NY, USA) were employed as feedstock material. However, it was amixture of Cr_3_C_2_ and Ni2OCr powders in a weight ratio of 75% and 25%. A scanning electron microscopy (SEM) image of the powder morphology and the grain size distribution is illustrated in Figure 1.

The Cr_3_C_2_ powder particles have an irregular shape, while the Ni20Cr particles have aspherical shape (Figure 1a). On the other hand, it is worth noting that in the Cr_3_C_2_-25(Ni20Cr) powder, the presence of alarge fraction of fine grains is noticeable (Figure 1b). Moreover, in order to minimize agglomeration effects, the powder was heated up to 110 °C in a convection oven for 1 h before use in thefeeder system.

**Figure 2:**
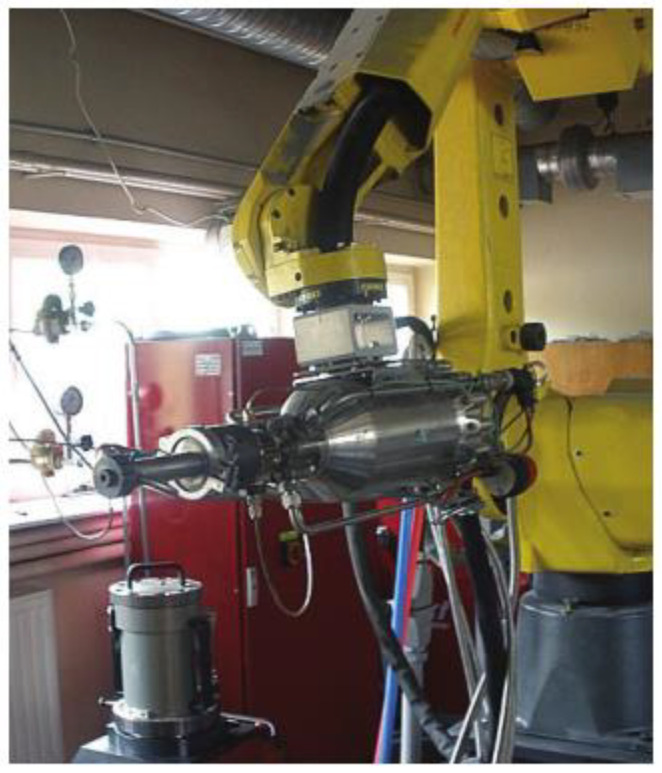
Cold gas spraying station.

For the production of the Cr_3_C_2_-25(Ni20Cr) cermet coating on the Al7075 substrate (Cr_3_C_2_–25(Ni20Cr)/Al7075) was used of cold gas spraying system Impact Innovations 5/8 equipped with the Fanuc M-20iA robot (Fanuc Robotics Ltd., Oshino, Japan), Figure 2.

The following parameters were used for the production ofcermet coatings: nitrogen pressure—30 bar; nitrogen preheating temperature—800 °C; spraying distance—60 mm, traverse speed—40 mm/s; the step size between 10 passes was 2 mm; the number of layers—4. The Cr_3_C_2_-25(Ni20Cr) cermet coatings were deposited on the Al7075 alloy. The surface of Al7075 substrates wasprepared by blasting with corundum of size 30 (600–710 μm). The specimen size was 310 × 110 × 5 mm^3^. The thicknesses of sprayed cermet coatings were in the range from 108 μm to 158 μm. However, the test specimens had the shape of a cuboid with dimensions of 30 × 10 × 5 mm^3^.

The cold gas sprayed coatings were laser remelted with three different velocities by means of a CO_2_ TRUMPF LASERCELL 1005 system (Trumpf GmbH, Ditzingen, Germany) appointed with a three-axial table. The laser remelting parameters are given in Table 1.

The measurement of microhardness of the tested materials was measured by the Vickers method (HV), using the Falcon 500 hardness tester from the INNOVATEST company (Maastricht, The Netherlands). An indenter was used in the form of a diamond pyramid with a square base, and an angle between opposite walls equal to 136° whose was load varied from 0.02 N to 20 N. The depth of indentation was about 2 μm.

The microstructure and cross-section of the specimens were observed by using a photo camera andscanning electron microscope (SEM) Joel (JEOL Ltd., Tokyo, Japan), type JSM-5400. The accelerating voltage of SEM was 20 kV. The chemical composition for the corroded surface was also measured by energy–dispersive spectrometer (EDS). Additionally, to observe the surface topography was used an inverted metallographic microscope (MO) IM-100 (Delta Optical, Warszawa, Poland). The topography of the coatings and the shape of the profile were examined using the Talysurf CCl-Lite 3D non-contact profilograph (Taylor Hobson Ltd., Leicester, UK).

**Figure 3:**
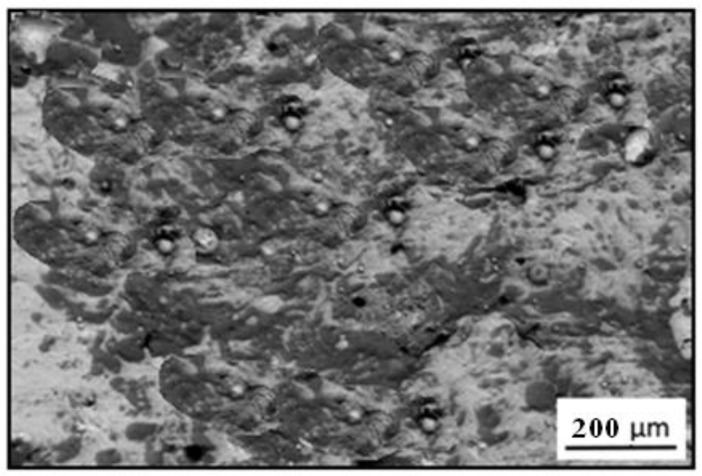
SEM image of the top surface of the Cr_3_C_2_-25(Ni20Cr) cermet coating on the Al7075 substrate.

Figure 3 shows the SEM image of the top surface of the Cr_3_C_2_-25(Ni20Cr) cermet coating on the Al7075 substrate.

The scanning electron microscope image of the cermet coating has indicated carbide particles (dark areas) in the metallic of the Ni-Cr matrix. However, the size of the carbides in the Cr_3_C_2_-25(Ni20Cr) layer did not exceed 3 μm.

X-ray diffraction (XRD) was applied to characterize the phase composition of powders and cold sprayed coatings before and after laser remelting using a Bruker D8 Discover diffractometer (Bruker Ltd., Malvern, UK), with Co Kα radiation of wavelength λ = 1.7889 Å.

The working electrode was made of the Cr_3_C_2_-25(Ni20Cr) cermet coating on the Al7075 substrate (Cr_3_C_2_-25(Ni20Cr)/Al7075). The geometric surface area of the working electrode was 1 cm^2^.

The saturated calomel electrode (SCE(KCl)) was used as the reference, and the counter electrode (5 cm^2^) was made of platinum foil (99.9% Pt).

The corrosion environment was obtained by mixing the sodium chloride and hydrochloric acid, so the concentration of Cl^−^ ion was 1.2 M. The pH value was 1.5.

The electrolyte was not deoxygenated.

The open-circuit potential (E_OCP_) in the corrosion environment was recorded within 60 min.

The potentiodynamic polarization (LSV) curves were recorded in the potential rangefrom −1000 mV to +200 mV vs. SCE(KCl), with a potential sweep of 1 mV/s. The LSV curves were used to designate the corrosion electrochemical parameters of the tested materials [21,22,23].

The chronoamperometric curves (ChA) were obtained for the potential values, which were selected on the basis of the potentiodynamic polarization curves.

All electrochemical measurements were carried out by using potentiostat/galvanostat, PGSTAT 128N (AutoLab, Amsterdam, Netherlands), piloted by NOVA 1.7 software.

All measurements were carried out at a temperature of 25 ± 0.5 °C, which were maintained using an air thermostat, home production.

## 3. Results and Discussion

### 3.1. Vickers Hardness of Material

The microhardness (HV) values of the Cr_3_C_2_-25(Ni20Cr) cermet coatings on the Al7075 substrate without and with laser remelting are listed in Table 2. The cermet coatings were produced by the cold sprayed method. However, the laser spot speed was 600 mm/min, 800 mm/min, and 1000 mm/min.

It was found that the laser remelting process changes the value of the surface hardness of the Cr_3_C_2_-25(Ni20Cr) cermet coating on the Al7075 substrate. Because using a laser spot that moved at 600 mm/min caused the hardness of the Cr_3_C_2_-25(Ni20Cr)/Al7075 surface to increase by about 60 HV10 units. It turned out that the surface hardness of the Cr_3_C_2_-25(Ni20Cr) cermet coatings for the higher values the laser spot speed (i.e., 800 mm/min, and 1000 mm/min) significantly decreased, reaching the value of 349 HV10 for the laser spot speed of 1000 mm/min (Table 2). Thus, the laser remelting of the Cr_3_C_2_-25(Ni20Cr) surface changes the structure of the alloy by smoothing and hardening the cermet coating, which is most pronounced for the laser spotspeed of 600 mm/min. It is worth adding that for the lower the laser spot speed values (i.e., 400 mm/min), a very deep melting of the Cr_3_C_2_-25(Ni20Cr) surface structure takes place, which causes deterioration of the mechanical properties of the cermet coating. On the other hand, for the high values of the laser spot speed (i.e., 1200 mm/min), no significant change in the value of the Cr_3_C_2_-25(Ni20Cr) surface hardness was observed.

### 3.2. Scanning Electron Microscopy Images

**Figure 4:**
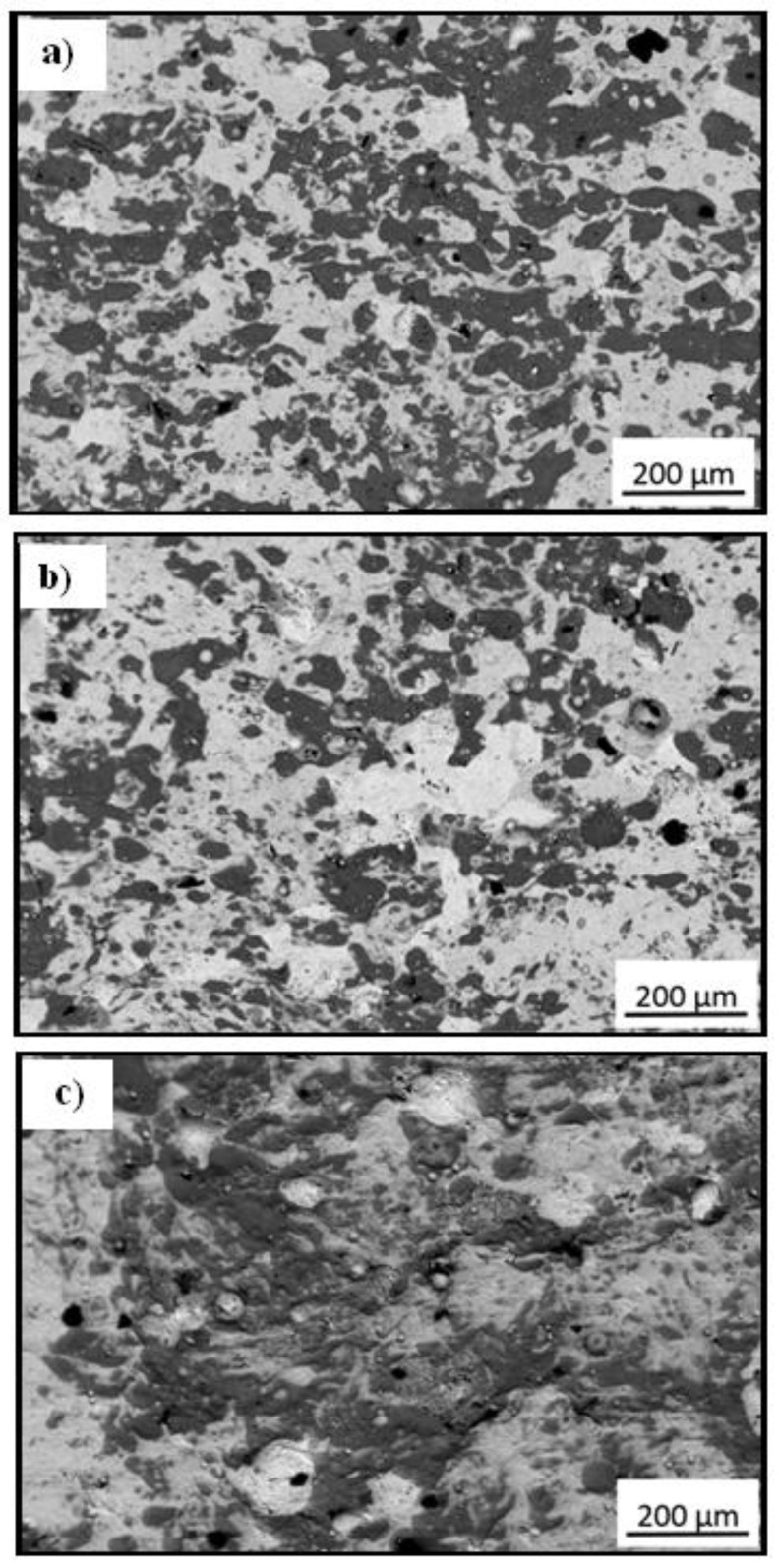
SEM imagesof the Cr_3_C_2_-25(Ni20Cr) cermet coatings on the Al7075 substrate after laser remelting for the spot speed: (**a**) 600 mm/min, (**b**) 800 mm/min, and (**c**) 1000 mm/min.

Figure 4 shows the scanning electron microscopy (SEM) imagesof the Cr_3_C_2_-25(Ni20Cr) cermet coatings on the Al7075 substrate after laser remelting for the different spot speeds.

**Figure 5:**
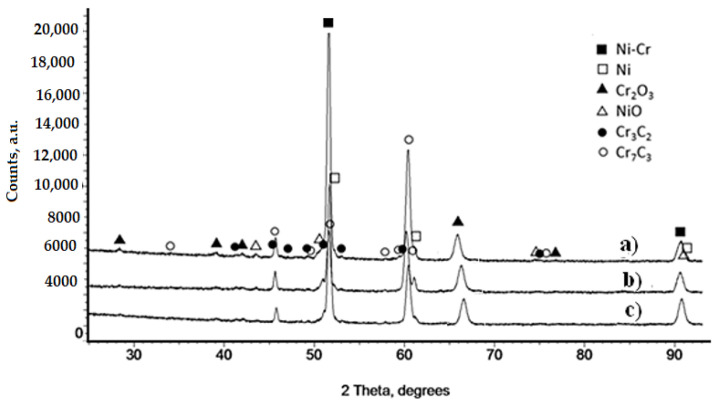
X-ray diffraction patterns obtained for the Cr_3_C_2_-25(Ni20Cr) cermet coatings on the Al7075 substrate after laser remelting for the spot speed: (**a**) 600 mm/min, (**b**) 800 mm/min, and (**c**) 1000 mm/min.

It was found that the laser spot speed has a significant influence on the surface structure of the Cr_3_C_2_-25(Ni20Cr)/Al7075. For the lowest, the laser spot speed, i.e., 600 mm/min, the flattest, regular, and compact cermet surface was obtained (Figure 4a). Moreover, for the larger the laser spot speed (800 mm/min, and 1000 mm/min), a rougher of the Cr_3_C_2_-25(Ni20Cr)/Al7075 surfaces were obtained (Figure 4b,c). However, the main component is the Ni20Cr matrix, which is the light phase. Additionally, new phases in the form of chromium and nickel oxides appeared on the surface of the coatings (Figure 4). A significant proportion of the surface is covered by the dark phase, which is chromium oxide [24]. This oxide was formed as a result of direct contact with the surrounding atmosphere despite the use of argon as a shielding gas. The appearance of this oxide on the surface cermet coatings was also reported by Matthews et al. [25]. Graphite, which is the black phase, is very clearly visible, distributed on the surface in the form of small rounded areas. However, when the temperature reaches the melting point of the matrix, the boundaries between deformed Ni20Cr grains disappeared in it. At the same time, fine carbide grains begin to dissolve in it and take on more round shapes. This is visible in the case of large chromium carbide grains that have retained a partially retained shape. The dissolution process produces fine grains of transformed Cr_3_C_2_ carbides that surround large grains [24]. The Cr_3_C_2_ carbide decomposed by peritectic reaction and Cr_7_C_3_ carbide under conditions of rapid cooling could probably then be formed. On the other hand, the laser melting process is a non-equilibrium process, and there are both types of chromium carbides in the melted layers. This analysis was confirmed by the carried-out investigations of the phase composition of all remelted coatings, which showed the presence of both chromium carbides in them, Figure 5.

The influence of the speed of the laser beam is visible; at the lowest speed of 600 mm/min, the heat input is the highest on the peaks of Cr_7_C_3_ are clearly higher, which means that more Cr_3_C_2_ was transformed into Cr_7_C_3_. Irregular spaces in the cold sprayed coatings filled with graphite become spherical. Moreover, the microstructure of remelted layers seems to be homogenous and independent of the laser spot speed.

**Figure 6:**
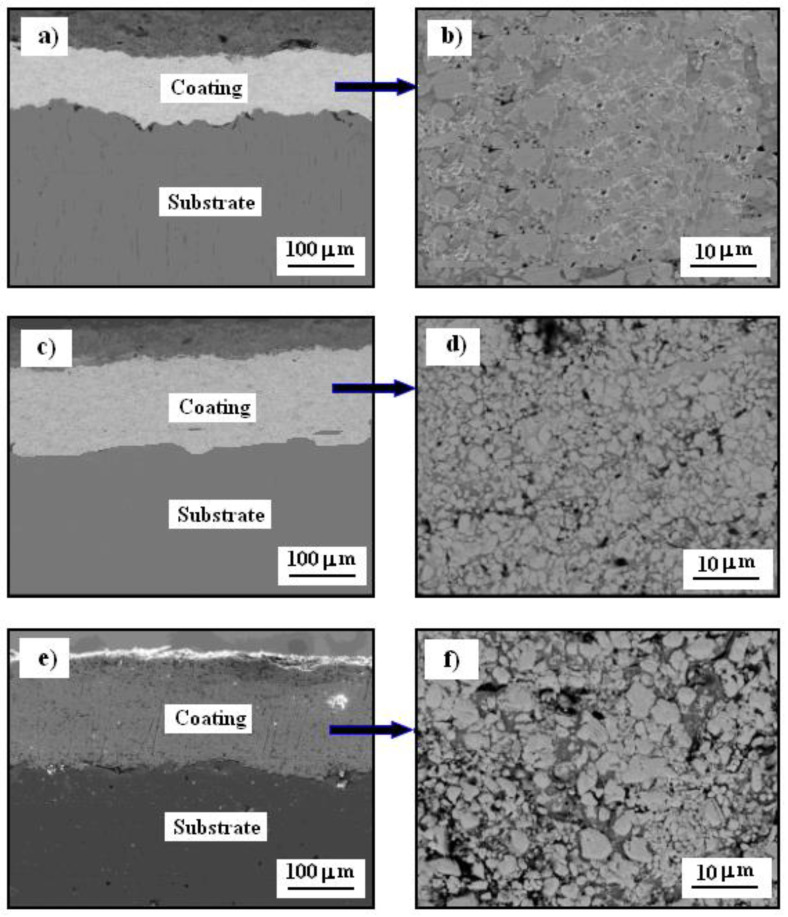
SEM cross-section imagesof the Cr_3_C_2_-25(Ni20Cr) cermet coatings on the Al7075 substrate after laser remelting for the spot speed: (**a**,**b**) 600 mm/min, (**c**,**d**) 800 mm/min, and (**e**,**f**) 1000 mm/min.

The scanning electron microscopy microstructure of the cross-section of the tested coatings is shown in Figure 6.

As a result of the laser treatment, the thickness of the Cr_3_C_2_-25(Ni20Cr) cermet coating was reduced. The lowest coating thickness was obtained for the laser spot speed of 600 mm/min (Figure 6a). The effect of a significant reduction in the thickness of the coating was obtained as a result of packing the structure of the tested coating. The obtained layer is very dense and homogenous because as a result of remelting the coating, the boundaries between the phases visible in the cold sprayed coating have disappeared (Figure 6b). Moreover, no cracks were observed in the obtained layer, which often occurs due to the relief of the accumulated thermal gradient stress during the laser remelting process. On the other hand, there is a significant visible change in the microstructure of the cold sprayed coatings after laser remelting for the spot speed of 800 mm/min or 1000 mm/min. As a result of the lower packing of the particles, the thickness of the cermet coatings on the Al7075 substrate is significantly greater (Figure 6c–e). The boundaries between the phases in both cermet shells are clearly visible (Figure 6d–f).

Therefore, it can be assumed that for the lowest speed of the laser spot (i.e., 600 mm/min), the Cr_3_C_2_-25(Ni20Cr) cermet coating was obtained, which should best protect the Al7075 substrate against contact with the corrosion environment.

### 3.3. Microstructure of Material

**Figure 7:**
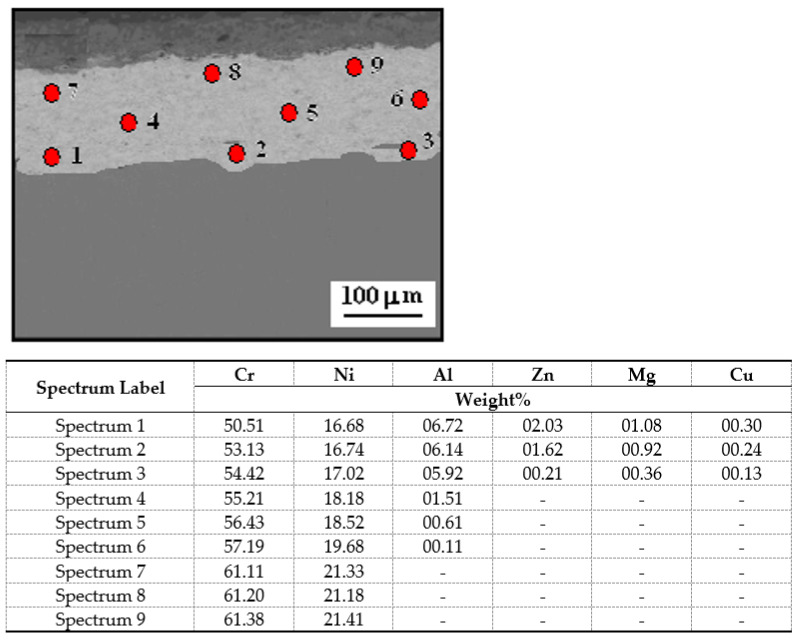
SEM image cross-section of the Cr_3_C_2_-25(Ni20Cr) cermet coating on the Al7075 substrate after laser remelting for the spot speed of 800 mm/min, and the results of point X-ray microanalysis of the chemical composition of the tested material.

Figure 7 depicts the SEM/EDS image of the cross-section of the Cr_3_C_2_-25(Ni20Cr) cermet coating on the Al7075 substrate with laser remelting coating for the spot speed of 800 mm/min, and the results of point X-ray microanalysis of the chemical composition of the tested material. Similar test results were obtained for the remaining laser spot speed, i.e., 600 mm/min and 1000 mm/min.

The average metal content (which are summarized in the Spectrum Label) in the Cr_3_C_2_-25(Ni20Cr) coatings was: 56.73%, 18.97%, and 3.50% for the elements Cr, Ni, and Al, respectively. The quantitative distribution of the elements along the cross-section of the coatings is not the same. The content of chromium and nickel increases systematically from the Al7075 substrate to the surface of the coatings. In addition, during the production of the Cr_3_C_2_-25(Ni20Cr) coatings by the cold sprayed method, aluminum and other elements (i.e., Zn, Mg, and Cu) permeated from the Al7075 substrate into the coatings.

**Figure 8:**
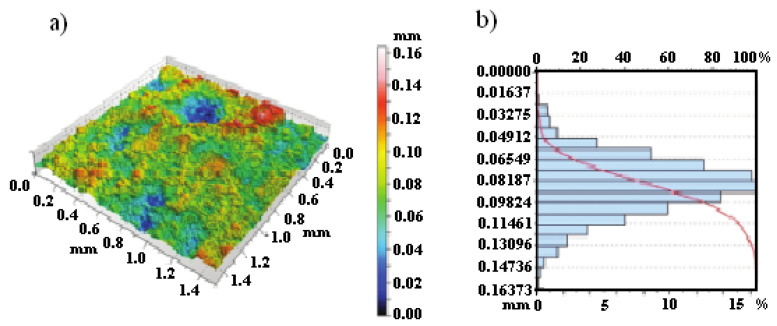
Structure of the Cr_3_C_2_-25(Ni20Cr) cermet coating on the Al7075 substrate after laser remelting for the spot speed of 600 mm/min: (**a**) coating topography, (**b**) depth histogram.

Figure 8 shows the surface topography and histogram roughness depth. The obtained results prove about high of the Cr_3_C_2_-25(Ni20Cr) surface roughness, which was produced by the cold gas method.

In this case, the surface roughness coefficient (*R*_a_) values were found to be within limits from 16.3 µm to 160.3 µm. On the other hand, for the higher the laser spot speed, the Ra values were much higher. Moreover, the tested coating has an asymmetric structure with anegative slope of the surface height. On the other hand, the value of the excess kurtosis was 3.2, which proves that the surface of the Cr_3_C_2_-25(Ni20Cr) cermet coating on the Al7075 substrate after laser remelting for the spot speed of 600 mm/min was free from extreme features of peaks and valleys. Therefore, the high roughness of the materials tested was due to the extensive grains diameter distribution that wasused to produce the Cr_3_C_2_-25(Ni20Cr) cermet coatings on the Al7075 substrate.

### 3.4. Corrosion Test

#### 3.4.1. Open-Circuit Potential Measurements

**Figure 9:**
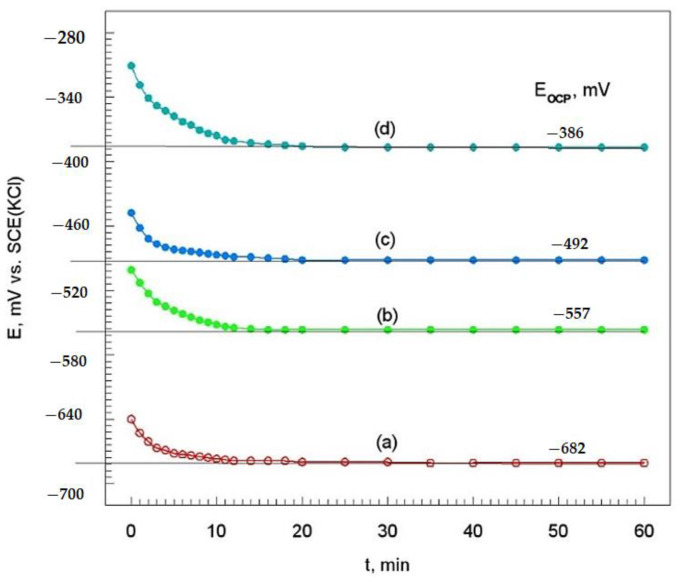
Open-circuit potential of the Cr_3_C_2_-25(Ni20Cr) cermet coatings on the Al7075 substrate: (**a**) before, and after laser remelting for the spot speed: (**b**) 1000 mm/min, (**c**) 800 mm/min, and (**d**) 600 mm/min. Solution contained 1.2 M Cl^−^, pH 1.5.

As the potential measured at each time was not locally measured, itonly represents an average potential value of all contributions of the sample in contact with the electrolyte and can provide information on the evolution and degrading of the coating. The open-circuit potential (E_OCP_) vs. time curves of the Cr_3_C_2_-25(Ni20Cr) cermet coatings on the Al7075 substrate are showed in Figure 9.

For all specimens, a slower change of potential was observed after ten minutes of immersion in the corrosion solution. By extrapolating the potential to zero time, the open-circuit potential values were determined of the Cr_3_C_2_-25(Ni20Cr) cermet coatings on the Al7075 substrate (Figure 9). It turned out that as the laser spot speed was decreased, the E_OCP_ values move towards positive values (i.e., from −682 mV to −386 mV vs. SCE(KCl)). Thus, it can be assumed that the Cr_3_C_2_-25(Ni20Cr) cermet coating on the Al7075 substrate becomes more resistant to electrochemical corrosion in the chloride environment for the lowest laser spot speed (i.e., 600 mm/min).

#### 3.4.2. Potentiodynamic Polarization Measurements

**Figure 10:**
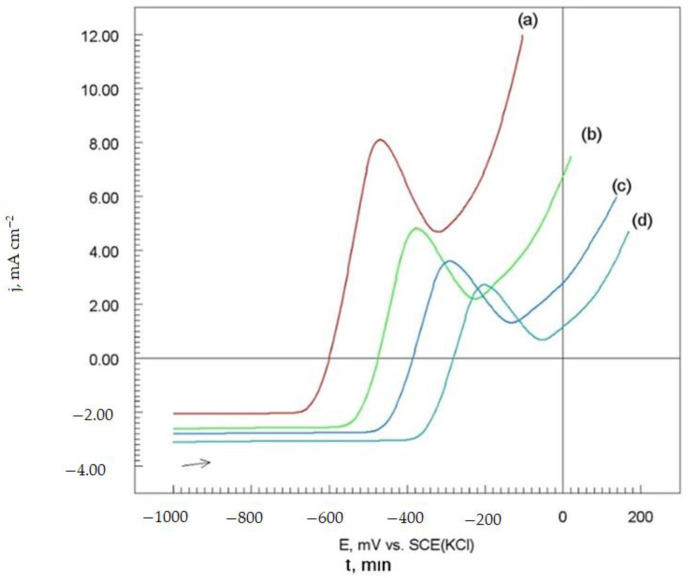
Potentiodynamic polarization curves of the Cr_3_C_2_-25(Ni20Cr) cermet coatings on the Al7075 substrate: (**a**) before, and after laser remelting for the spot speed: (**b**) 1000 mm/min, (**c**) 800 mm/min, and (**d**) 600 mm/min. Solution contained 1.2 M Cl^−^, pH 1.5, d*E*/d*t* 1 mV/s.

Potentiodynamic polarization (LSV) measurements were carried out in order to gain knowledge concerning the impact of laser remelting on the anti-corrosion properties of the Cr_3_C_2_-25(Ni20Cr) cermet coating on the Al7075 substrate and kinetics of the cathodic and anodic reactions. Figure 10 shows potentiodynamic polarization curves of the Cr_3_C_2_-25(Ni20Cr)/Al7075, before and after laser remelting for the different spot speeds, i.e., 600 mm/min, 800 mm/min, and 1000 mm/min.

The process of hydrogen depolarization occurs in the cathode region of the potentiodynamic polarization curves. In the acid corrosion environment, the cathodic branches of the LSV curves correspond to the simplified reduction of hydrogen ions [21,22,23]:

Me^0^ + n H^+^→Me^0^ + n H_2_ − m e^−^ (2)

where Me means the Cr, Ni, and other metals.

The oxidation process (anode region) of the surface of the Cr_3_C_2_-25(Ni20Cr) cermet coating on the Al7075 substrate depends on the laser spot speed. The shift of the LSV curves towards the positive potentials may indicate an increase in the corrosion resistance of the Cr_3_C_2_-25(Ni20Cr) cermet coatings, especially for the lowest laser spot speed, i.e., 600 mm/min (Figure 10, curve (d)). Moreover, when Al7075 was covered with Cr_3_C_2_-25(Ni20Cr) cermet coating, the anodic reaction was as follows [21,22,23]:

Me^0^ + 2 H^+^ + O_2_ (MeO)_ads_ H_2_O + m e^−^ (3)

where (MeO)_ads_ means (Cr_2_O_3_)_ads_, (NiO)_ads_, and other oxides. In this case, the working electrode surface was covered mainly with a layer of (Cr_2_O_3_)_ads_, (NiO)_ads_ oxides. However, oxides adhered well to the electrode surface. Thus, the Cr_3_C_2_-25(Ni20Cr) coating was passivated under the experimental conditions.The characteristic peaks related to the passivation process of the Cr_3_C_2_-25(Ni20Cr) coating appeared in the LSV curves. Passivation peaks are observed in a wide range of electrode potential, i.e., from −510 mV to −190 mV vs. SCE(KCl)) for the tested materials (Figure 10). Therefore, a clear inhibition of the corrosion process of the protective coating was observed.

It seems that under these conditions, the adsorbed oxide layer can be additionally sealed by adsorption of Cl^−^ ions [22]:

(MeO)_ads_ + Cl^−^ + H^+^ → (MeClOH)_ads_ (4)

The adsorption layer (MeClOH)_ads_ in the acidic chloride solution weredissolved in accordance with a chemical reaction [21,22,23]:

(MeClOH)_ads_ + H^+^ → Me^n+^ + Cl^−^ + H_2_O (5)

Thus, a further sharp increase in the current intensity is observed due to the oxidation of the electrode surface (Figure 10). However, for a more positive electrode potential of the Cr_3_C_2_-25(Ni20Cr), coatings were depassivated, and further oxidation of the tested materials was observed (Figure 10, curves (a)–(d)). This problem will be discussed extensively later in the article.

#### 3.4.3. Corrosion Electrochemical Parameters

The potentiodynamic polarization curves (Figure 10) were used to designate the corrosion parameters of the tested materials, i.e., Cr_3_C_2_-25(Ni20Cr) cermet coatings on the Al7075 substrate before and after laser remelting. For this purpose, the method of the extrapolation of rectilinear sections of the Tafel LSV curves wasused [21,22,23]. The values of the corrosion parameters of the tested materials are listed in Table 3.

The corrosion potential (E_corr_) of the investigated materials has shifted significantly towards the positive values compared to the Cr_3_C_2_-25(Ni20Cr) cermet coating on the Al7075 substrate without laser treatment. This means that the use of laser remelting increases the corrosion resistance of Cr_3_C_2_-25(Ni20Cr)/Al7075 in an acid chloride solution. It seems that the most corrosion-resistant coating was obtained for the lowest laser spot speed, i.e., 600 mm/min (Table 3). However, as the laser spot speed decreases, the slope of the cathodic (−b_c_) and anodic (b_a_) sections of the potentiodynamic polarization curves systematically decreases in a narrow range (about 100 mV). Therefore, the change of the laser spot speed does not significantly change the mechanism of the cathode and anode process on the surface of the Cr_3_C_2_-25(Ni20Cr) cermet coating. It is worth noting that the corrosion current density (j_corr_) systematically decreases as the laser spot speed slows down (Table 3). It can be assumed that for the laser spot speed, i.e., 600 mm/min, the Cr_3_C_2_-25(Ni20Cr) cermet coating will have the lowest rate of electrochemical corrosion in aggressive chloride environments.

#### 3.4.4. Polarization Resistance and Corrosion Rate

In order to determine the values of the polarization resistance (Rp) of the Cr_3_C_2_-25(Ni20Cr) cermet coatings without and with laser treatment in an aggressive chloride environment, fragments of potentiodynamic polarization curves (Figure 10), which relate to the active dissolution area of the tested materials were selected. The polarization resistance of the electrode is described by the equation [21,22,23]:

(6) $$R_{p}=\frac{B}{j_{\mathrm{corr}}}$$

and:

(7) $$B=\frac{b_{a}\times b_{c}}{2.303\left(b_{a}+b_{c}\right)}$$

The R_p_ valuesdepending on the laser spot speed are summarized in Table 4.

It was found that the polarization resistance of the Cr_3_C_2_-25(Ni20Cr)/Al7075 electrode surface increases as the laser spot speed decreases (Table 4). Moreover, the polarization resistance of the Cr_3_C_2_-25(Ni20Cr) laser, treated with the spot speed of 600 mm/min, doubled compared to the Cr_3_C_2_-25(Ni20Cr)/Al7075,which was not subjected to laser irradiation.Thus, in the case of the protective coating irradiated with the laser spot speed of 600 mm/min, the mass and electric charge exchange between the Cr_3_C_2_-25(Ni20Cr) electrode and the chloride electrolyte solution was difficult.

The corrosion rate of the Cr_3_C_2_-25(Ni20Cr) cermet coatings on the Al7075 substrate without and with laser treatment was calculated based on the equation:

CR (mm/year) = 1.16 j_corr_ (8)

which by authors [26,27,28] was proposed. The values of the corrosion rate of the Cr_3_C_2_-25(Ni20Cr) cermet coatings are listed in Table 5.

The laser spot speed of the cermet coating has a significant influence on the values of the corrosion rate (CR) of the Cr_3_C_2_-25(Ni20Cr) coatings on the Al7075 substrate. It turned out that for the laser spot speed of 600 mm/min, the lowest CR, i.e., 0.46 mm/year, was observed for the Cr_3_C_2_-25(Ni20Cr)/Al7075. However, for a laser spot speed of 1000 mm/min, the corrosion rate of the Cr_3_C_2_-25(Ni20Cr) cermet coating is more than twice as high as the CR of the tested coatings for the lowest laser spot speed (Table 5).

### 3.5. Chronoamperometric Measurements

**Figure 11:**
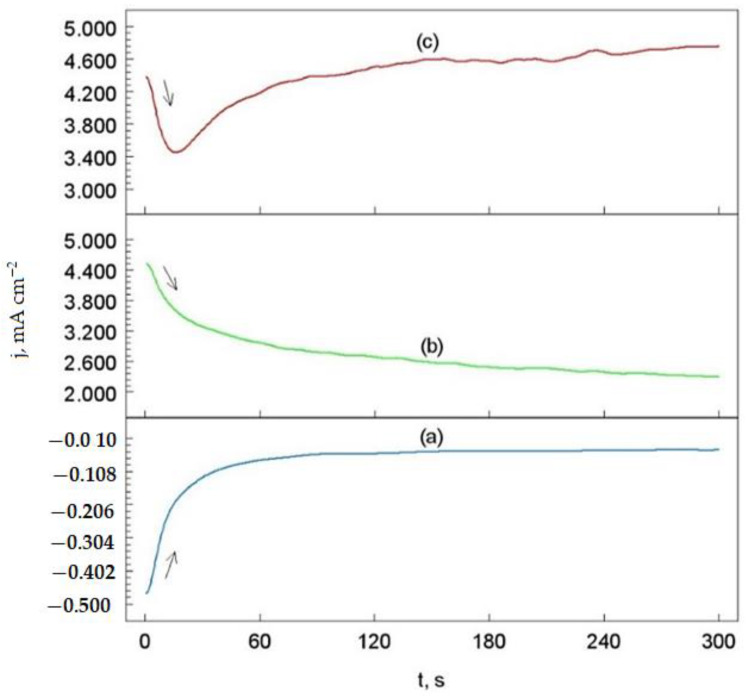
Chronoamperometric curves of the Cr_3_C_2_-25(Ni20Cr) cermet coating on the Al7075 substrate after laser remelting for the spot speed of 600 mm/min, obtained for: (**a**) −900 mV, (**b**) −190 mV, and (**c**) −40 mV. Solution contained 1.2 M Cl^−^, pH 1.5.

Figure 11 shows the chronoamperometric (ChA) curves of the Cr_3_C_2_-25(Ni20Cr) cermet coating on the Al7075 substrate after laser remelting for the spot speed of 600 mm/min. However, similar ChA curves were obtained for the remaining laser spot speed but are not quoted in this work. All curves were recorded in the acid chloride (1.2 M Cl^−^) environment.

The potentials of the working electrode were selected based on the potentiodynamic polarization curve (Figure 10, curve (d)), i.e., for the laser spot speed of 600 mm/min. However, for the potential of −900 mV vs. SCE(KCl), the H^+^ ions reduction process (reaction (2) took place on the surface of the working electrode (Figure 11, curve (a)).

On the other hand, for the potentials of −190 mV and −40 mV vs. SCE(KCl), oxidation of the surface of the Cr_3_C_2_-25(Ni20Cr) cermet coating was observed (reactions (3)–(5)). It is worth noting that for the peak potential of −190 mV, the oxidation current density of the electrode material systematically decreases with the passage of electrolysis time (Figure 11, curve (b)). Thus, during the electrolysis process, as a result of the reaction (3), the oxide layer on the Cr_3_C_2_-25(Ni20Cr)/Al7075 surface was sealed due to the adsorption of (Cr_2_O_3_)_ads_ and (NiO)_ads_ oxides. Moreover, the adsorbed layer of nickel, chromium oxides, and other element oxides could be additionally sealed in the form of the adsorbed of (MeClOH)_ads_ layer (reaction (4)) on the Cr_3_C_2_-25(Ni20Cr)/Al7075 surface.

In the case of a more positive potential of the working electrode (i.e., −40 mV), the oxidation current density of the Cr_3_C_2_-25(Ni20Cr)/Al7075 surface initially decreases (up to 20 s) and then increases with increasing electrolysis time (Figure 11, curve (c)). This means that the protective layer adsorbed on the surface of the working electrode in the acid chloride solution was partially dissolved. Thus, the exposureof the Cr_3_C_2_-25(Ni20Cr) coating was corroded in the aggressive corrosion environment of chlorides (reaction (5)).

### 3.6. Photographic Images after Corrosion Test

**Figure 12:**
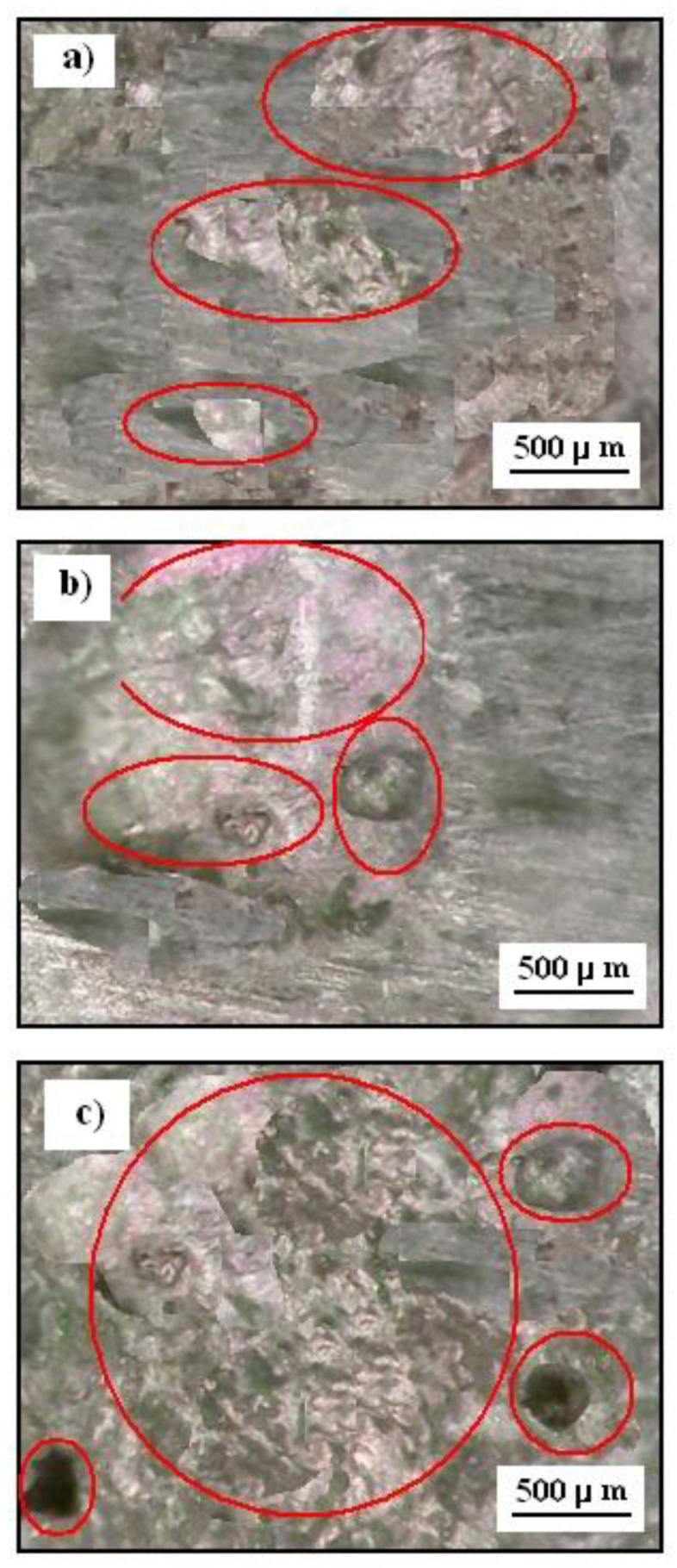
MO images of the Cr_3_C_2_-25(Ni20Cr) cermet coatings on the Al7075 substrate after laser remelting for the spot speed: (**a**) 600. mm/min, (**b**) 800 mm/min, and (**c**) 1000 mm/min in 1.2 M Cl^−^, pH 1.5. Exposure time was five hours. (Corrosion damages are marked in red).

Figure 12 shows the inverted metallographic microscope (MO) images of the Cr_3_C_2_-25(Ni20Cr) cermet coatings on the Al7075 substrate after laser remelting for the spot speed of 600 mm/min, 800 mm/min, and 1000 mm/min. The Cr_3_C_2_-25(Ni20Cr)/Al7075 surface was subjected to the corrosion test in an acid chloride solution (1.2 M Cl^−^). The exposure time of the specimen was five hours. However, the oxide layer from the surface of the tested specimen was removed with diluted nitric acid. In this case, the exposure time was about three minutes.

The surfaces of the Cr_3_C_2_-25(Ni20Cr) cermet coatings were subject to corrosion as aresult of long contact with a strong electrolyte. As a result of the corrosion process, numerous pits appeared on the surface of all samples, which reduced the mechanical and aesthetic properties of the tested materials (Figure 12). The lowest corrosion damage was observed for the Cr_3_C_2_-25(Ni20Cr)/Al7075 surface, which was subjected to laser processing with alaser spot speed of 600 mm/min (Figure 12a). In this case, the Cr_3_C_2_-25(Ni20Cr) coating was hardened and sealed as a result of the laser treatment, and the corrosion process of the investigated material was significantly slowed down. Thus, the Cr_3_C_2_-25(Ni20Cr) coating protects the Al7075 substrate well against contact with a corrosion environment, i.e., 1.2 M Cl^−^.

On the other hand, for the higher laser spot speed, i.e., 800 mm/min or 1000 mm/min were much greater damage to the Cr_3_C_2_-25(Ni20Cr)/Al7075 surfaces due to corrosion in the aggressive environment of chloride (Figure 12b,c).

## 4. Conclusions

The influence of the laser treatment on the corrosion resistance of the Cr_3_C_2_-25(Ni20Cr) cermet coating on the Al7075 substrate was investigated. The Cr_3_C_2_-25(Ni20Cr)/Al7075 coating was produced by the cold sprayed (CS) method. The speed of the laser remelting of the Cr_3_C_2_-25(Ni20Cr) was varied from 600 mm/min to 1000 mm/min. Laser remelting has a significant influence on the surface structure of the cermet coatings. For the lowest speed (i.e., 600 mm/min), the flattest, regular, and compact cermet surface on the Al7075 substrate was obtained. However, the Vickers microhardness of the cermet coatings was decreased as the speed of laser irradiation was increased. The highest polarization resistance (R_p_) was observed for the Cr_3_C_2_-25(Ni20Cr) coating after remelting for the speed of 600 mm/min. Therefore, in this case, the lowest corrosion rate (CR) of the Cr_3_C_2_-25(Ni20Cr) surface in the chloride environment was recorded. The protective, homogeneous oxide layer was formed on the Cr_3_C_2_-25(Ni20Cr) surface, which very effectively protects the tested materials against corrosion. Moreover, for the higher speeds, laser remelting (i.e., 800 mm/min or 1000 mm/min) significantly reduces the mechanical and anti-corrosion properties of the cermet coatings.

## Figures and Tables

**Table 1:** Laser remelting parameters.

Parameter	Value
Power, kW	6
Protective gas	Argon
Laser spot speed, mm/min	600, 800, 1000
Spot size, mm	10 × 1

**Table 2:** Vickers microhardness of the Cr_3_C_2_-25(Ni20Cr) cermet coatings on the Al7075 substrate.

Laser Spot Speed mm/min	HV10
Without laser remelting	326 ± 4
1000	349 ± 2
800	361 ± 3
600	384 ± 3

**Table 3:** Corrosion electrochemical parameters of the Cr_3_C_2_-25(Ni20Cr) cermet coatings on the Al7075 substrate without and with laser remelting.

Laser Spot Speed mm/min	E_corr_ mV vs. SCE(KCl)	−b_c_	b_a_	j_corr_ mA/cm^2^
		mV/dec		
Without laser remelting	−598	170	130	1.80
1000	−472	130	90	0.90
800	−385	90	80	0.60
600	−279	70	60	0.40

**Table 4:** Polarization resistance of the Cr_3_C_2_-25(Ni20Cr) cermet coatings on the Al7075 substrate without and with laser remelting.

Laser Spot Speed mm/min	R_p_ mΩ cm^2^
Without laser remelting	17.8
1000	25.6
800	30.6
600	35.1

**Table 5:** Corrosion rate of the Cr_3_C_2_-25(Ni20Cr) cermet coatings on the Al7075 substrate without and with laser remelting.

Laser Spot Speed mm/min	CR mm/year
Without laser remelting	2.08
1000	1.04
800	0.70
600	0.46

## Data Availability

Data sharing not applicable.
